# Teratogenic Effects and Outcomes of Congenital Oropouche Virus Infection: Current Evidence, Pathophysiological Mechanisms, and Research Priorities

**DOI:** 10.3390/v18101101

**Published:** 2026-10-05

**Authors:** Léo Pomar, Loïc Epelboin, Najeh Hcini

**Affiliations:** 1 Ultrasound and Fetal Medicine Unit, Department Woman-Mother-Child, Lausanne University Hospital and University of Lausanne, 1011 Lausanne, Switzerland; 2 HESAV School of Health Sciences-Vaud, HES-SO University of Applied Sciences and Arts Western Switzerland, 1022 Chavannes-près-Renens, Switzerland; 3 Unité des Maladies infectieuses et Tropicales, Centre d’Investigation Clinique-INSERM 1424, CH de Cayenne Andrée Rosemon-Université de Guyane, 97300 Cayenne, French Guiana; 4 Department of Obstetrics and Gynaecology, French Guiana University Hospital, 97320 Saint-Laurent-du-Maroni, French Guiana; 5 Santé des populations en Amazonie, Université de Guyane, DFR Santé, 97300 Cayenne, French Guiana

**Keywords:** Oropouche virus, pregnancy, congenital infection, vertical transmission, microcephaly, arboviruses, teratogenicity, neurodevelopment

## Abstract

Background: Oropouche virus (OROV), an Orthobunyavirus endemic to Latin America, has recently emerged as a pathogen of concern in pregnancy following reports of vertical transmission, fetal demise, congenital anomalies, and neonatal disease. The unprecedented 2023–2025 outbreaks in Latin America have expanded the geographic distribution of OROV and renewed concerns regarding its potential as an emerging human teratogen. Methods: We conducted a narrative comprehensive review of the literature focusing on epidemiology, maternal–fetal transmission, placental infection, experimental models, fetal and neonatal outcomes, and comparisons with other congenital arboviral infections. Results: Increasing evidence supports the ability of OROV to infect the placenta, cross the maternal–fetal interface, and invade fetal tissues. Human cases have documented miscarriage, stillbirth, fetal demise, microcephaly, ventriculomegaly, cerebral atrophy, corpus callosum abnormalities, posterior fossa defects, arthrogryposis, and neonatal death following maternal infection. Viral RNA and OROV-specific antibodies have been detected in placental, fetal, and neonatal samples, providing direct evidence of congenital infection. Experimental studies using trophoblast cultures, placental organoids, neural progenitor cells, brain organoids, and animal models have confirmed placental susceptibility, vertical transmission, fetal neurotropism, and disruption of neurodevelopment. The congenital phenotype shares important similarities with congenital Zika syndrome and other neurotropic arboviral infections. Conclusions: OROV is newly and increasingly recognized as a vertically transmissible neurotropic arbovirus with potential teratogenic effects. Although biological plausibility is supported by converging clinical and experimental findings, prospective epidemiological studies are urgently needed to quantify risks, identify determinants of fetal injury, and define long-term outcomes among affected children.

## 1. Introduction

Oropouche virus (OROV), recently recalled *Orthobunyavirus oropoucheense*, is an emerging arthropod-borne virus belonging to the genus *Orthobunyavirus* within the family *Peribunyaviridae* [1,2,3,4,5]. Since its first identification in Trinidad and Tobago in 1955, OROV has become one of the most important causes of arboviral febrile illness in Latin America, with recurrent outbreaks reported in Brazil, Peru, Ecuador, Bolivia, and other countries of the Amazon basin [3,4].

Historically, OROV infection was considered a self-limiting febrile illness characterized by fever, headache, myalgia, arthralgia, and occasional neurological manifestations. However, large outbreaks reported since 2023 in South America and the Caribbean have renewed interest in the virus. This attention has been further heightened by emerging evidence suggesting adverse pregnancy outcomes after maternal infection [2,6,7].

The recognition of congenital infections associated with OROV has drawn inevitable comparisons with the emergence of congenital Zika virus (ZIKV) syndrome during the 2015–2016 pandemic. Reports of fetal demise, microcephaly, structural brain abnormalities, and detection of viral RNA in fetal tissues have strengthened concerns regarding the teratogenic potential of this virus [2,8,9,10,11].

Simultaneously, advances in experimental animal research have demonstrated that OROV infects placental tissues, replicates in trophoblasts, crosses the maternal–fetal interface, and targets fetal neural cells, providing mechanistic support for the clinical observations [12,13,14,15,16].

This review summarizes current knowledge regarding congenital OROV infection, including epidemiology, pathophysiological mechanisms, placental transmission, fetal and neonatal outcomes, and similarities and differences with other congenital arboviral infections. Beyond summarizing the available evidence, we aim to integrate emerging clinical and experimental findings into a unified framework and identify critical knowledge gaps and priorities for future research.

## 2. Materials and Methods

A comprehensive narrative review was conducted using publications indexed in PubMed, Scopus, Web of Science, Embase, and Google Scholar through June 2026. Additional relevant reports from the World Health Organization (WHO), Pan American Health Organization (PAHO), and Centers for Disease Control and Prevention (CDC) were reviewed.

Search terms included combinations of: “Oropouche virus”, “pregnancy”, “vertical transmission”, “congenital infection”, “microcephaly”, “birth defects”, “placenta”, “trophoblast”, “brain organoid”, and “teratogenicity”.

Original studies, case reports, case series, epidemiological investigations, experimental research, and review articles relevant to maternal–fetal infection were included.

## 3. Results

### 3.1. Transmission

OROV circulates through both sylvatic and urban transmission cycles. The primary urban vector is the biting midge *Culicoides paraensis*, although several mosquito species, including *Culex quinquefasciatus*, *Coquillettidia venezuelensis*, and *Aedes serratus*, have also been implicated in enzootic transmission cycles [4,5,6]. In the sylvatic cycle, a variety of vertebrate hosts, including birds, rodents, non-human primates, and sloths, are considered potential reservoirs. Arthropod vectors acquire infection by feeding on viremic animals, thereby maintaining virus circulation in forest environments. In urban settings, humans are believed to act as the principal amplifying hosts, facilitating sustained transmission during outbreaks [17,18].

Recent outbreaks have demonstrated an expanding geographic distribution, potentially driven by urbanization, environmental and climate changes, deforestation, and increasing human mobility [3,5,7,19]. Although OROV RNA has been detected in the semen of infected individuals, evidence supporting sexual transmission remains lacking, and vector-borne transmission continues to be regarded as the dominant route of human infection.

### 3.2. Epidemiology of OROV Infection and Pregnancy Exposure

Since its identification in 1955, OROV has been responsible for more than 30 recognized outbreaks throughout Latin America, with Brazil accounting for most reported cases [3,4,5]. Cumulative estimates suggest that more than 500,000 human infections have occurred since the virus was first identified, making OROV one of the most important neglected arboviruses in the Americas [3,4]. However, the true burden of disease remains uncertain because OROV infection is frequently misdiagnosed as dengue, chikungunya, Zika, or other acute febrile illnesses. Underdiagnosis is further compounded by the limited availability of testing in endemic regions [3,5]. The epidemiology of OROV changed substantially during the 2023–2025 outbreak, which represents the largest epidemic recorded since the virus was first identified. According to the Pan American Health Organization (PAHO), 16,239 laboratory-confirmed cases were reported in 2024 across 11 countries and one territory in the Americas, including four deaths reported in Brazil. Between epidemiological week 1 and week 30 of 2025, a further 12,786 confirmed cases were reported from 11 countries, bringing the cumulative number of reported cases during the 2024–2025 outbreak to more than 29,000. Although Brazil accounted for most infections (13,785 cases in 2024 and 11,888 cases in 2025), sustained transmission was also documented in Bolivia, Cuba, Peru, Panama, Colombia, Guyana, and Venezuela, while imported cases were reported in North America and Europe [20].

**Figure 1:**
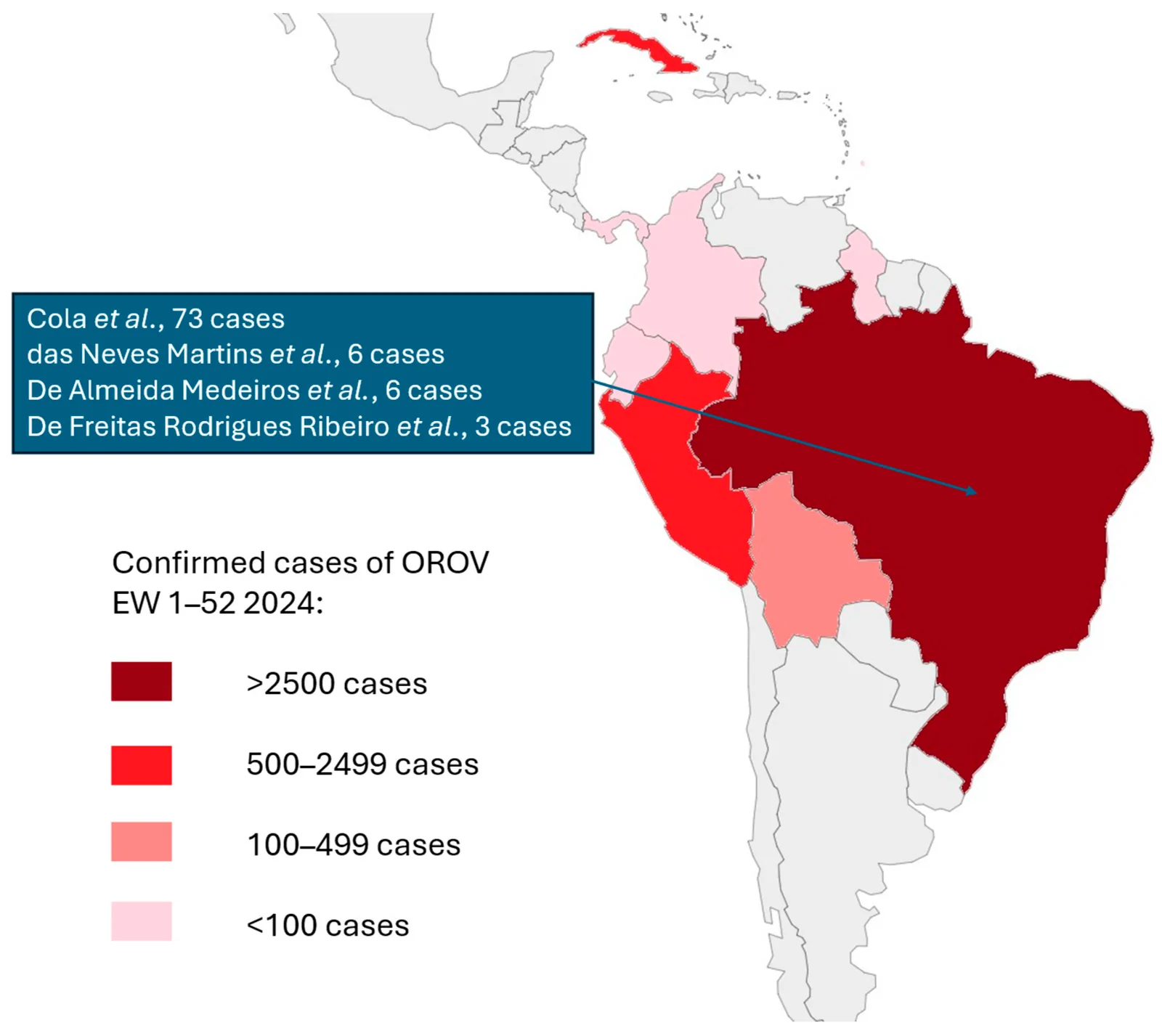
Geographic distribution of confirmed autochthonous Oropouche virus (OROV) cases in South America and the Caribbean during the 2024 outbreak and reported pregnancy outcomes. Countries were categorized according to the cumulative number of confirmed autochthonous OROV cases reported between epidemiological week 1 to 52, 2024: dark red (>2500 cases), red (500–2500 cases), light red (100–499 cases), and pink (<100 cases). The studies reporting pregnancy outcomes presented the following recruitment periods: das Neves Martins et al. [June 2016–June 2024] [9], Cola et al. [March–December 2024] [8], De Almeida Medeiros et al. [May–August 2024] [10], and De Freitas Rodrigues Ribeiro et al. [April–July 2024] [11]. Data on OROV incidence was extracted from the PAHO/WHO Epidemiological Update of August 2025 [20].

During the 2023–2025 resurgence, surveillance systems in Brazil identified the first fatal cases and clusters of adverse pregnancy outcomes. Reported pregnancy outcomes included spontaneous abortions, stillbirths, and congenital anomalies following maternal infections that occurred during the 2024 outbreak [2,6,8,9,10,11] (Figure 1). The largest pregnancy cohort reported to date included 73 pregnant women with confirmed OROV infection in Espírito Santo State, Brazil, during 2024. Among the 15 pregnancies with available outcomes, 14 resulted in live births and one in spontaneous abortion, while one of two documented first-trimester infections was associated with corpus callosum dysgenesis in the newborn [8].

The increasing recognition of adverse pregnancy outcomes during the 2024 outbreak has renewed interest in understanding the biological mechanisms underlying maternal–fetal transmission and fetal injury. Experimental studies have therefore become essential for investigating the pathogenesis of congenital OROV infection.

### 3.3. Experimental Models and Mechanisms of OROV Vertical Transmission and Fetal Injury

Experimental models have substantially advanced the understanding of OROV pathogenesis and tissue tropism. Early studies in murine and hamster models demonstrated systemic viral dissemination with preferential involvement of the liver and central nervous system (CNS), resulting in hepatocellular injury, neuroinvasion, motor impairment, paralysis, and meningoencephalitis [21]. The host immune response appears to play a crucial role in controlling infection. OROV induces both innate and adaptive immune responses, including production of virus-specific IgM and IgG antibodies and activation of type I interferon pathways. Experimental studies have demonstrated that type I interferon signaling is a major determinant of resistance to placental and fetal infection, with impaired interferon responses facilitating vertical transmission and severe fetoplacental disease [15,21]. At the cellular level, OROV exhibits broad cellular tropism. In vitro studies have shown productive infection of dendritic cells, CD4-positive T lymphocytes, monocytes, and peripheral blood mononuclear cells, suggesting that immune cells may contribute to viral dissemination throughout the host [21]. More recently, human placental explants and trophoblast organoid models demonstrated efficient infection and replication within cytotrophoblasts and syncytiotrophoblasts at the maternal–fetal interface, providing biological evidence supporting transplacental transmission [4,5]. Accumulating evidence also indicates that OROV possesses substantial neurodevelopmental pathogenic potential. Human neural progenitor cells are highly permissive to infection, and infected brain organoids exhibit impaired growth, increased cell death, and microcephaly-like phenotypes [12,13,14]. Current evidence supports a pathogenic model in which maternal viremia is followed by placental infection, viral replication within trophoblastic tissues, transplacental dissemination, and direct invasion of fetal organs, particularly the developing brain. Placental injury, dysregulated immune responses, and infection of neural progenitor cells likely act synergistically to produce the spectrum of congenital abnormalities currently associated with congenital OROV infection [4,5,12,13,14,15,16]. These experimental findings provide biological plausibility for the occurrence of congenital infection and fetal injury. However, understanding the clinical presentation of OROV infection in pregnant women and its diagnosis remains essential to identify exposed pregnancies and contextualize their outcomes.

### 3.4. Maternal Symptoms and Outcomes

**Figure 2:**
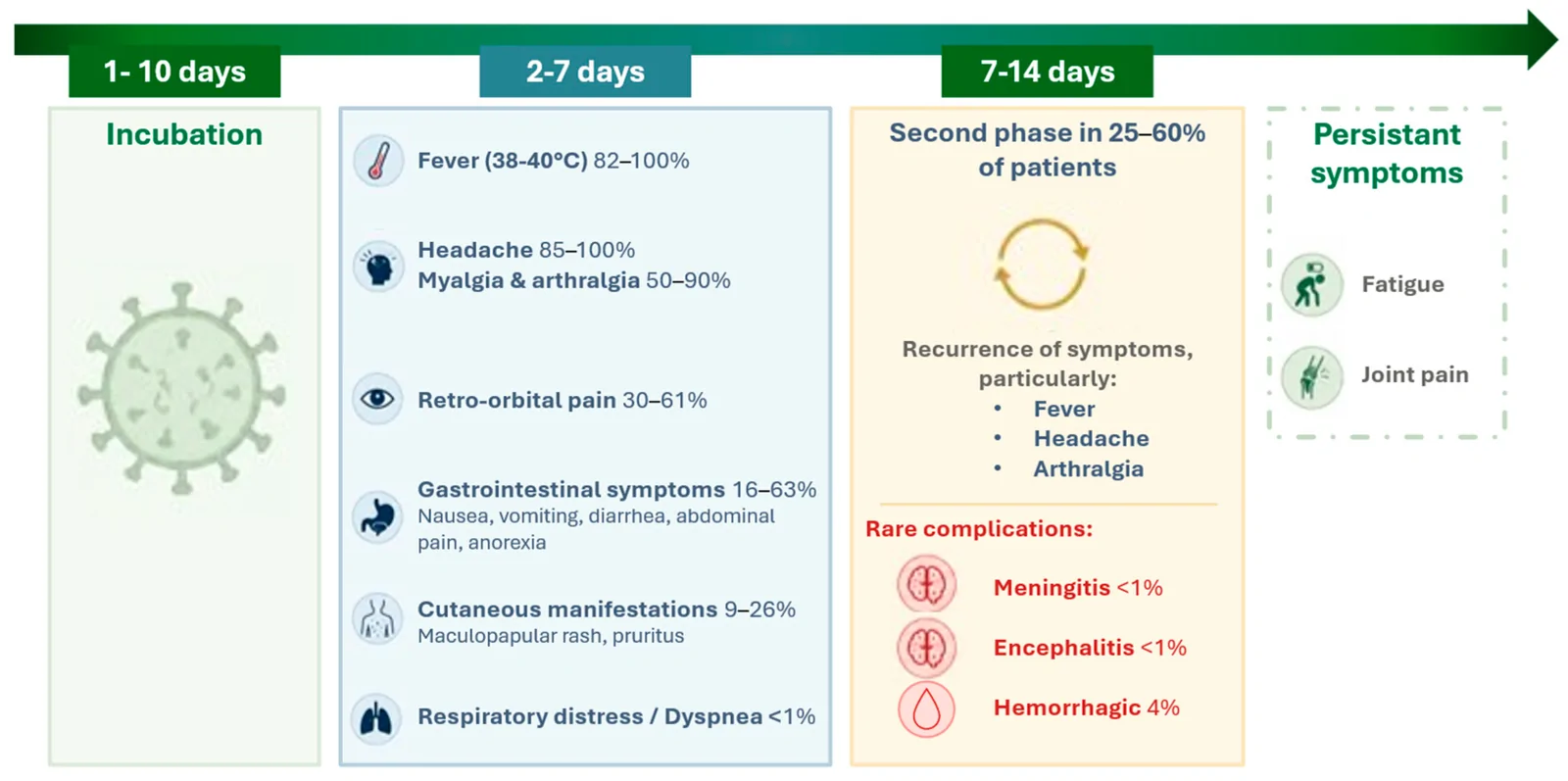
Clinical course and temporal evolution of Oropouche virus (OROV) infection. Following an incubation period of approximately 1–10 days, symptomatic disease typically presents with an acute febrile phase lasting 2–7 days, characterized by high fever (38–40 °C), severe headache, diffuse myalgia, migratory arthralgia, gastrointestinal symptoms (nausea, vomiting, abdominal discomfort, diarrhea, and anorexia), and occasional retro-orbital pain. Transient cutaneous manifestations, including pallor, pruritus, and maculopapular rash, may occur, while respiratory symptoms such as cough, sore throat, nasal congestion, and dyspnea are uncommon. Frequency ranges are derived from published epidemiological studies and meta-analysis [22,23,26,27,28,29,30,31]. A biphasic clinical course is reported in 25–60% of patients [3,21,23,24,25] with recurrence of fever, headache, and arthralgia occurring 7–14 days after the initial illness. Neurological complications, including meningitis, encephalitis or hemorrhagic complications, are rare [19,26,30,32]. Persistent symptoms, most commonly fatigue and joint pain, may continue beyond the acute phase in a subset of patients.

The clinical spectrum of OROV infection ranges from asymptomatic infection to acute febrile disease and, more rarely, severe neurological or systemic complications. Following an incubation period of approximately one week (1–10 days), symptomatic individuals typically present with a sudden onset of fever (pooled prevalence 94%, 95% CI 90–98%), accompanied by headache (87%, 95% CI 84–91%), myalgia (73%, 95% CI 67–80%), arthralgia (51%, 95% CI 41–62%), chills (49%, 95% CI 26–72%), and fatigue or weakness (49%, 95% CI 21–77%) [22]. Retro-orbital pain is also commonly reported (43%, 95% CI 35–52%). Because these manifestations overlap considerably with those of dengue, chikungunya, Zika, and other arboviral diseases, clinical diagnosis alone remains unreliable in areas where multiple arboviruses co-circulate [21,22]. Gastrointestinal manifestations are frequent and include loss of appetite (41%, 95% CI 28–55%), nausea or vomiting (36%, 95% CI 31–42%), abdominal pain (18%, 95% CI 9–27%), and diarrhea (20%, 95% CI 13–26%) [22]. Other reported symptoms include dizziness (35%, 95% CI 21–49%) and photophobia (33%, 95% CI 12–54%). Cutaneous manifestations, such as rash (16%, 95% CI 9–23%) and pruritus (17%, 95% CI 8–26%), are less common and lack diagnostic specificity. Likewise, respiratory symptoms, including sore throat (29%, 95% CI 21–38%), may occur but are not considered defining features of the disease [21,22]. Acute illness is usually self-limited, with symptom resolution occurring within several days. However, recurrent symptoms have been described in a substantial proportion of patients, resulting in a biphasic clinical pattern characterized by renewed fever, headache, and arthralgia 7–14 days after apparent recovery [3,21,23,24,25]. While most infections remain uncomplicated, severe manifestations have occasionally been reported. Neurological complications, including meningitis, encephalitis, and meningoencephalitis, are rare, with pooled prevalences below 1%, whereas hemorrhagic manifestations occur in approximately 4% of reported cases (95% CI 0–7%) [22,26]. During recent outbreaks, rare cases of hepatic dysfunction, renal impairment, and fatal disease have also been described [21,27]. The clinical course of symptomatic OROV infections and pooled frequency ranges of symptoms and complications are summarized in Figure 2.

Most reported infections during pregnancy appear clinically similar to those observed in non-pregnant adults [21]. At present, there is little evidence that pregnancy substantially modifies the maternal clinical phenotype or increases susceptibility to severe disease [2,6,7,8,9,10,11]. However, systemic inflammatory responses to OROV infection may contribute to preterm labor, hypertensive disorders of pregnancy, or maternal organ dysfunction, although higher rates for these outcomes have not been documented yet. Thus, from an obstetric perspective, the primary concern is not maternal morbidity itself, but the potential for mother-to-child transmission and associated adverse pregnancy outcomes, including miscarriage, stillbirth, congenital anomalies, fetal distress, and the subsequent need for obstetric interventions.

### 3.5. Maternal Infection Diagnosis and Testing Considerations

Diagnosis of maternal OROV infection is challenging because clinical manifestations overlap substantially with those of other arboviral infections, such us dengue, chikungunya, Zika virus infection or other tropical infections such as malaria, leptospirosis and *Coxiella burnetii* infection (Q fever) [21]. Consequently, laboratory confirmation is essential whenever OROV infection is suspected during pregnancy. Reverse-transcription polymerase chain reaction (RT-PCR) is currently the preferred diagnostic method during the early acute phase of maternal infection (5 to 7 days) [21,33]. Modern RT-qPCR assays have demonstrated good performances, with reported amplification efficiencies exceeding 98% and limits of detection ranging from 2 to 20 viral copies per reaction, making them highly suitable for diagnosing acute infection [33]. OROV RNA can be detected by RT-PCR in several biological specimens, including blood, urine, saliva, and cerebrospinal fluid (CSF). After the acute viremic phase, diagnosis relies primarily on serological methods. OROV-specific IgM antibodies typically become detectable 5 days after symptom onset and may persist beyond the period of detectable viremia [11,21]. IgM-capture ELISA assays have reported sensitivity of approximately 93% and specificity approaching 99% in research settings, but their interpretation may be complicated by antibody cross-reactivity with other co-circulating arboviruses [34]. Plaque reduction neutralization testing (PRNT) remains the most specific serological method for confirming OROV infection and differentiating OROV from other arboviral infections in cases with suspected cross-reactivity [35,36,37,38]. Emerging recombinant antigen-based immunoassays based on recombinant OROV nucleoprotein and glycoprotein antigens have shown promising diagnostic performance in preliminary studies, although further clinical validation is required before their widespread implementation [39].

Routine clinical implementation is limited by the need for paired serum samples, specialized laboratory facilities, and complex technical procedures. Accordingly, a combined diagnostic approach using RT-PCR during the acute phase and serological testing during convalescence currently represents the most practical strategy for maternal diagnosis [21]. In the absence of established recommendations for systematic OROV screening during pregnancy, testing for OROV can be considered in pregnant women presenting with an unexplained acute febrile illness during or following travel to, or residence in, an area with ongoing or recent OROV transmission. Importantly, OROV infection should also be considered when ultrasound features suggestive of fetal infection are identified in areas with ongoing or recent OROV transmission, particularly when the standard first-line work-up for congenital infections is negative. Similarly, unexplained fetal death or stillbirth in an endemic or epidemic area, or after returning from an area at risk for OROV transmission (particularly the Amazon Basin and other affected regions of Brazil, Peru, Bolivia, Colombia, Panama, Cuba, and neighboring countries), should prompt consideration of OROV testing as part of the etiological work-up. Because fetal manifestations may become apparent only after a variable interval following maternal infection, the absence of a documented maternal febrile episode should not necessarily exclude OROV from the differential diagnosis.

### 3.6. Vertical Transmission of Oropouche Virus

Evidence of vertical transmission in humans is supported by the detection of OROV RNA in placental tissue, umbilical cord blood, cerebrospinal fluid, and multiple fetal organs obtained from affected pregnancies [2,8,9,10,11]. In severe cases, viral RNA has been identified in the fetal brain, lungs, kidneys, heart, liver, and spleen, with some studies reporting the highest viral loads in neural tissue, consistent with the pronounced neurotropism observed in experimental models [9,10,11]. Additional support for congenital infection comes from maternal–newborn investigations demonstrating OROV-specific IgM antibodies in neonatal serum and cerebrospinal fluid, indicating an active fetal immune response following intrauterine exposure [9,11,21]. The strongest epidemiological evidence currently originates from Brazil, where clusters of miscarriages, stillbirths, fetal deaths, microcephaly, and congenital malformations were identified during the recent outbreak [6,8,9,10,11,20]. In Espírito Santo State, OROV RNA was detected by RT-PCR in placental samples from infected pregnancies, providing direct evidence of placental infection and transplacental viral passage [8]. The same cohort also reported a newborn with early neonatal symptoms and detectable OROV RNA shortly after birth, suggesting that intrapartum transmission may occur in addition to classical transplacental infection [8].

Experimental studies provide strong biological plausibility for these clinical observations. Human placental explants, trophoblast cultures, and trophoblast organoids have been shown to support productive viral replication, confirming that placental tissues are permissive to OROV infection [4,5]. Furthermore, type I interferon signaling appears to be a critical determinant of protection at the maternal–fetal interface, as disruption of this pathway markedly increases placental infection, fetal dissemination, and vertical transmission in experimental systems [15]. Animal models have further strengthened the evidence base. Murine studies have demonstrated that OROV efficiently replicates in maternal tissues, crosses the maternal–fetal interface, and infects both placental and fetal compartments, resulting in fetal growth restriction, congenital abnormalities, and fetal loss [12,15,16]. Importantly, similar findings have been observed with both historic and recently circulating strains, suggesting that vertical transmission is likely an intrinsic characteristic of OROV biology rather than a property limited to contemporary outbreak variants [40]. Consistent with these observations, infection of human neural progenitor cells and forebrain organoids results in impaired neurodevelopment, increased cellular injury, and microcephaly-like phenotypes, providing mechanistic support for the congenital abnormalities reported in humans [12,13,14].

Despite increasingly compelling evidence, major uncertainties remain. Current knowledge is largely derived from case reports, case series, surveillance investigations, and experimental models, whereas prospective epidemiological studies remain lacking [1,21]. Consequently, the true incidence of vertical transmission, the determinants of fetal susceptibility, and the absolute risk of congenital disease following maternal infection remain unknown. Given the scarcity of available data, no evidence-based recommendation can currently be made regarding the indication for amniocentesis in pregnant women with OROV infection or the diagnostic value of testing amniotic fluid for OROV RNA. In contrast, the epidemiology of congenital ZIKV syndrome has been clarified through large prospective pregnancy cohorts and pooled analyses, which enabled robust estimation of transmission rates and adverse fetal outcomes [41,42]. Building upon lessons learned from the ZIKV epidemic, several authors have emphasized the urgent need for coordinated surveillance systems, including prospective pregnancy cohorts [43,44].

### 3.7. Congenital Oropouche Virus Infections and Fetal/Neonatal Outcomes

The clinical spectrum of congenital OROV infection remains incompletely defined but seems to extend from asymptomatic infection to severe fetal and neonatal disease [8,9,10,11]. Pregnancy loss appears among the most frequently reported outcomes, including first-trimester miscarriage, intrauterine fetal demise, and stillbirth [2,6,8,10,21]. Among surviving fetuses and neonates, abnormalities predominantly involve the central nervous system and include microcephaly, ventriculomegaly, cerebral atrophy, porencephalic cysts, corpus callosum agenesis or dysgenesis, posterior fossa abnormalities, cerebellar hypoplasia, and destructive cerebral lesions identified by prenatal imaging, postnatal assessment, or pathological examination [9,10,11,45]. Reported extracerebral manifestations include arthrogryposis, joint contractures, microphthalmia, and hepatosplenomegaly, suggesting that congenital infection may represent a multisystem disorder rather than an exclusively neurotropic syndrome, and should prompt a monthly prenatal ultrasound follow-up including neurosonography, in case of maternal exposure [10,11,21,46,47,48]. Although available data remain limited, the timing of maternal infection and subsequent fetal exposure is likely to be a major determinant of disease severity, as observed with other congenital viral infections. The most severe outcomes reported to date, including miscarriage, fetal demise, microcephaly, and major structural brain abnormalities, have largely followed infections diagnosed during the first and second trimesters [6,8,9,10,11,21]. During these critical periods of embryogenesis and fetal brain development, viral infection may disrupt key processes such as neural progenitor proliferation, neuronal migration, cortical organization, and synaptogenesis [12,13,14]. This hypothesis is consistent with both experimental OROV models and observations from congenital Zika syndrome, where early gestational infection is associated with the highest risk of severe neurological sequelae [41,49,50,51,52].

The fetal phenotype observed following congenital OROV infection shares remarkable similarities with that reported in other neuroteratogenic arboviral infections (Table 1).

**Figure 3:**
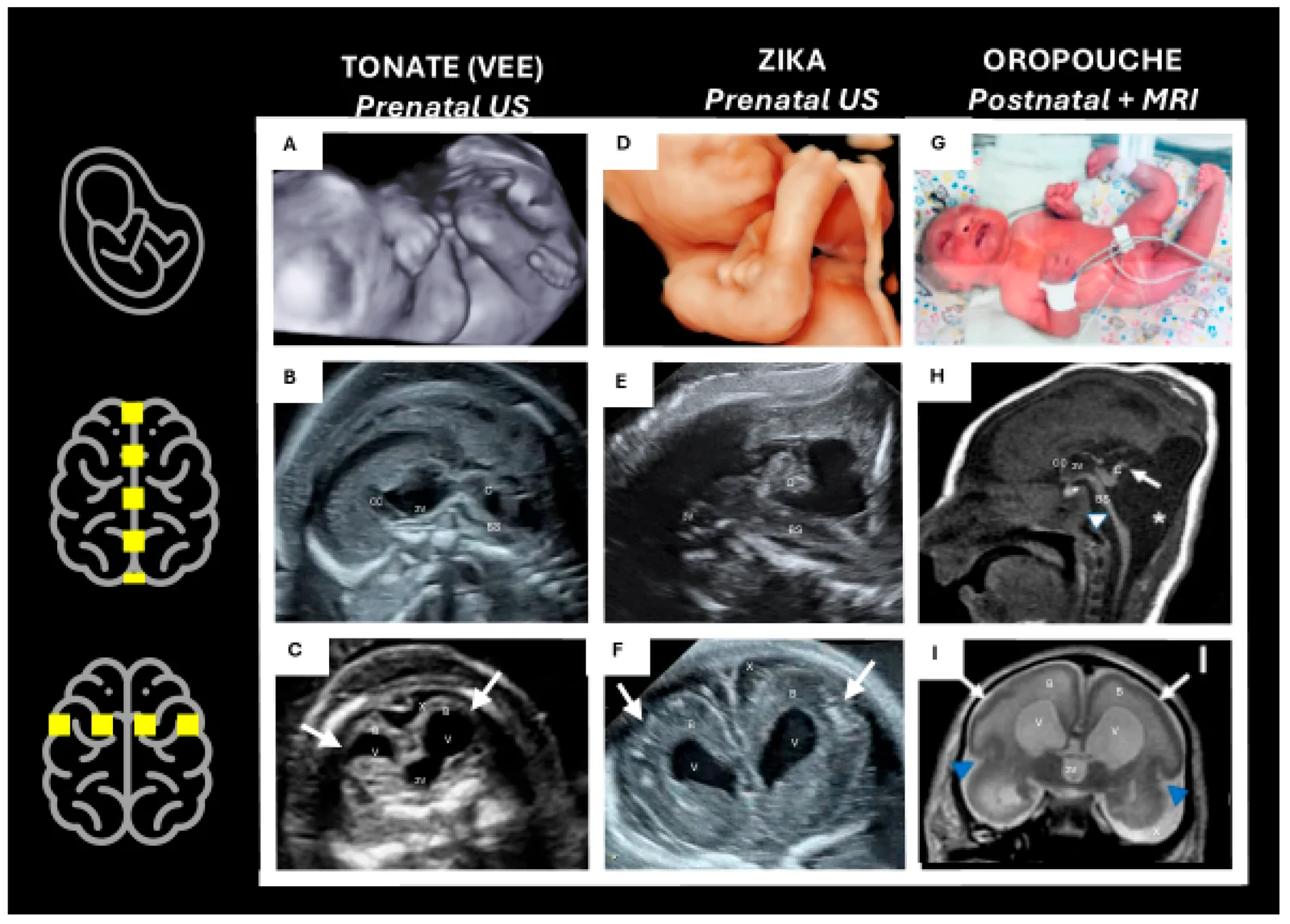
Shared malformative spectrum of congenital Oropouche, Zika, and Tonate (Venezuelan equine encephalitis complex) virus infections. Prenatal ultrasound and postnatal examination and MRI in case of congenital Tonate syndrome at 21 weeks’ gestation (**A**–**C**), congenital Zika syndrome at 28 weeks’ gestation (**D**–**F**), and postnatal congenital Oropouche syndrome (**G**–**I)** showing: arthrogryposis (**A**,**D**,**G**), and brain, cerebellar and brainstem (BS) atrophy associated with enlarged extracerebral spaces (X), ventriculomegaly, malformations of the cortical development on fetal neurosonography and postnatal MRI in the midsagittal (**B**,**E**,**H**), and coronal (**C**,**F**,**I**) planes. Anatomical planes are indicated to left of images. Images presented have been adapted from the original publication [11,61]; 3V, third ventricle; B, brain parenchyma; C, cerebellum; CC, corpus callosum; V, lateral ventricles; X, extra-cerebral spaces; *, enlarged fourth ventricle.

Several of the cerebral abnormalities described in OROV-exposed fetuses, including severe cortical atrophy, ventriculomegaly, destructive cerebral lesions, corpus callosum abnormalities, posterior fossa involvement, arthrogryposis, and microcephaly, overlap with the spectrum observed in congenital Zika syndrome [41,49,50,51,52,59,60]. Similar patterns of fetal brain injury have also been documented following congenital infection with Tonate virus, a member of the Venezuelan equine encephalitis complex, where fetal cortical destruction, microcephaly, ventriculomegaly, and cerebral atrophy were reported [53,57,58]. Furthermore, in 2002, congenital West Nile virus infection has been associated with severe fetal encephalopathy and destructive cerebral abnormalities, suggesting that several neuroinvasive arboviruses may share common pathogenic mechanisms during fetal development [54,55,56]. Although the number of documented cases remains limited, these observations raise the possibility that OROV belongs to a broader group of congenital neurotropic arboviral infections characterized by placental infection, fetal neuroinvasion, impaired neural progenitor development, and a recurrent pattern of cortical atrophy, cystic cerebral destruction, ventriculomegaly, microcephaly, intracranial calcifications, and arthrogryposis [61] (Figure 3). Future comparative studies may help determine whether congenital OROV infection represents a distinct clinical syndrome or forms part of a broader spectrum of arbovirus-associated fetal brain injury.

Neonatal manifestations of congenital OROV infection may extend beyond structural anomalies identified prenatally. Reported live-born infants have presented with severe microcephaly, marked craniofacial disproportion, hypotonia, feeding difficulties, impaired swallowing, and severe motor dysfunction associated with arthrogryposis and joint contractures [11,21]. Ocular abnormalities, including chorioretinal lesions and pigmentary retinal changes, have also been described, further supporting the multisystem nature of congenital infection [11]. In the most severe cases, neonatal or early infant death has occurred following extensive neurological and systemic involvement [9,11]. Although long-term follow-up data are currently lacking, available clinical observations and experimental models raise concerns regarding persistent neurodevelopmental sequelae among surviving children. Similar to congenital Zika syndrome, adverse outcomes may not be fully apparent at birth and could emerge during infancy through impaired head growth, developmental delay, motor dysfunction, epilepsy, visual impairment, hearing abnormalities, or cognitive deficits [43,60,62]. This concern is strengthened by experimental studies showing profound disruption of neural progenitor proliferation, cerebral growth restriction, and microcephaly-like phenotypes in organoid and animal models [12,13,14,16]. Consequently, all infants with confirmed or suspected congenital OROV exposure should undergo long-term multidisciplinary follow-up, including neurological, ophthalmological, audiological, and developmental assessments, even in the absence of overt neonatal abnormalities [21,44].

### 3.8. Diagnosis of Congenital Oropouche Virus Infection

The diagnosis of congenital OROV infection is challenging because neither prenatal imaging findings nor neonatal clinical manifestations are pathognomonic. Structural abnormalities associated with congenital OROV infection, including microcephaly, ventriculomegaly, cerebral atrophy, posterior fossa abnormalities, corpus callosum defects, and arthrogryposis, overlap with those observed in other congenital infections, particularly congenital Zika syndrome and TORCH (Toxoplasmosis, Others, Rubella, Cytomegalovirus, Herpes simplex virus) pathogens [21,61]. Consequently, laboratory confirmation is required whenever maternal OROV infection is suspected during pregnancy or when compatible fetal or neonatal abnormalities are identified in areas with documented viral circulation [1,21]. Current recommendations emphasize a multimodal diagnostic approach combining maternal testing during the acute phase, targeted fetal assessment, and comprehensive neonatal evaluation [21].

For maternal infection, RT-PCR remains the preferred diagnostic method during the first week after symptom onset, whereas serological assays are most informative thereafter [8,21,33]. In pregnancies complicated by confirmed or suspected maternal infection, serial ultrasound examinations including dedicated neurosonography are recommended, with particular attention to cerebral biometry, cortical development, ventriculomegaly, posterior fossa structures, and fetal growth [46,47,48]. When fetal abnormalities are detected, amniotic fluid testing through amniocentesis may be considered in selected cases, although its diagnostic performance remains unknown [21].

After delivery, diagnostic investigations should include maternal blood, placental tissue, umbilical cord blood, and neonatal blood. Additional samples such as cerebrospinal fluid, amniotic fluid, fetal tissues, or postmortem specimens should be obtained when congenital infection is suspected [2,21]. Demonstration of OROV RNA or viral antigens within placental, fetal, or neonatal tissues currently provides the strongest evidence supporting congenital infection [2,21] (Table 2).

## 4. Discussion

The findings summarized in this review provide converging epidemiological, clinical, pathological, and experimental evidence supporting the emergence of OROV as a potential congenital pathogen. The following discussion evaluates the strength of this evidence using established teratogenicity frameworks.

### 4.1. Does the Current Evidence Support a Teratogenic Role for OROV?

The accumulation of clinical, pathological, and experimental evidence over the past two years has substantially strengthened the hypothesis that OROV is capable of causing congenital infection and fetal injury. Although the number of documented human cases remains limited, the convergence of epidemiological observations, virological findings, placental investigations, and experimental models supports a causal relationship between maternal infection and adverse fetal outcomes [2,8,9,10,11,21,63]. Traditionally, teratogenic causality has been assessed using criteria derived from Shepard and complementary epidemiological frameworks such as the Bradford Hill criteria [64,65]. While OROV does not yet fulfill all requirements necessary for classification as a confirmed human teratogen, several key criteria are already satisfied.

### 4.2. Evaluation According to Shepard’s Criteria

Several of Shepard’s criteria for proof of human teratogenicity appear to be fulfilled, at least partially [65]. Exposure during critical periods of embryogenesis has been documented in multiple affected pregnancies [8,9,10,11,21]. A relatively consistent pattern of anomalies has emerged across independent reports, predominantly involving the central nervous system, including microcephaly, ventriculomegaly, cerebral atrophy, corpus callosum abnormalities, posterior fossa abnormalities, and arthrogryposis. Furthermore, maternal–fetal transmission has been demonstrated through the detection of viral RNA and virus-specific antibodies in placental, fetal, and neonatal tissues [2,8,9,10,11,21]. However, important criteria remain unmet. In particular, no large prospective cohort has yet quantified the risk of congenital disease following maternal infection, and no robust epidemiological estimate of relative risk is currently available [43,44,63]. Consequently, the available evidence is highly suggestive but remains insufficient to establish OROV as a proven human teratogen according to classical teratological standards [63,65].

### 4.3. Evaluation According to Bradford Hill’s Criteria

Application of Bradford Hill’s criteria also supports a causal interpretation of the association between maternal OROV infection and fetal disease [64]. Temporality is fulfilled, as maternal infection consistently precedes fetal abnormalities, fetal demise, or neonatal manifestations [2,8,9,10,11,45]. Biological plausibility is strongly supported by evidence of placental infection, transplacental dissemination, and direct infection of neural progenitor cells and developing neural tissues [4,5,12,13,14,15,16]. Consistency is demonstrated by the convergence of findings from independent case reports, surveillance studies, pathological investigations, and experimental models [2,8,11]. Coherence is reinforced by the remarkable agreement between the lesions observed in affected fetuses and those reproduced experimentally in placental, organoid, and animal models [12,13,14,15,16]. The criterion of analogy is particularly compelling. Other neurotropic arboviruses, including ZIKV, West Nile virus (WNV), and members of the Venezuelan equine encephalitis complex, have previously been shown to cause congenital infection and severe fetal neurological injury [41,42,43,61]. The similarities highlighted in Figure 2 therefore strengthen the biological plausibility of OROV-associated congenital disease. By contrast, the criteria of strength of association, dose–response relationship, and population-level reproducibility cannot yet be adequately assessed due to the absence of prospective cohorts and reliable incidence estimates [43,44,63].

### 4.4. Knowledge Gaps and Future Directions

Despite rapidly increasing evidence, major uncertainties remain. The true incidence of vertical transmission, the absolute risk of congenital disease following maternal infection, and the frequency of long-term neurodevelopmental sequelae remain unknown [43,44,63]. Likewise, the relative contributions of direct viral injury, placental dysfunction, and maternal immune responses to fetal pathogenesis have yet to be fully elucidated [4,5,12,13,14,15,16]. Lessons learned from the ZIKV epidemic clearly demonstrate the importance of coordinated surveillance networks, standardized case definitions, prospective pregnancy cohorts, biobanking of maternal–fetal specimens, and long-term follow-up of exposed infants [43,44,50]. Similar efforts will be required to determine whether OROV should ultimately be classified as a confirmed human teratogen and whether a distinct congenital Oropouche syndrome can be formally established [61].

## 5. Conclusions

OROV has recently emerged as a significant concern in maternal–fetal medicine. Although historically regarded as a self-limited arboviral infection, accumulating evidence now demonstrates the capacity of OROV to infect the placenta, cross the maternal–fetal interface, and cause congenital infection. Clinical reports, pathological investigations, and experimental studies consistently support a causal link between maternal infection and a spectrum of adverse fetal outcomes, including miscarriage, fetal demise, and severe neurodevelopmental abnormalities. The congenital phenotype associated with OROV infection is characterized predominantly by central nervous system involvement, including microcephaly, ventriculomegaly, cerebral atrophy, destructive cerebral lesions, corpus callosum abnormalities, and posterior fossa defects. Notably, these findings overlap substantially with those observed in other congenital neurotropic arboviral infections, particularly congenital Zika syndrome, and may indicate the existence of shared pathogenetic mechanisms involving placental infection, fetal neuroinvasion, and disruption of early brain development. While current evidence fulfills several important criteria supporting teratogenic causality, significant knowledge gaps remain. The incidence of vertical transmission, the magnitude of fetal risk following maternal infection, and the long-term neurodevelopmental consequences among surviving children remain unknown. Future priorities should include prospective pregnancy cohorts, standardized surveillance systems permitting systematic investigation of fetal and neonatal cases, and long-term follow-up of exposed infants. Such efforts will be essential to fully characterize congenital OROV infection, quantify its burden, and determine whether a distinct congenital Oropouche syndrome can be formally established.

## Figures and Tables

**Table 1:** Comparison of congenital manifestations and available evidence across selected neurotropic arboviral infections.

Characteristics Reported	OROV	ZIKV	WNV	VEE
Evidence of vertical transmission	Yes	Yes	Yes	Yes
Estimated rate of vertical transmission	No	26%	4%	No
Placental infection	Yes	Yes	Yes	Yes
Pregnancy loss (miscarriage, stillbirth)	Yes	Yes	Yes	Yes
Microcephaly	Yes	Yes	Yes	Yes
Ventriculomegaly	Yes	Yes	Yes	Yes
Cerebral atrophy/destructive lesions	Yes	Yes	Yes	Yes
Corpus callosum abnormalities	Yes	Yes	Yes	Yes
Arthrogryposis	Yes	Yes	No	Yes
Ocular abnormalities	Yes	Yes	Yes	Yes

The table summarizes current evidence regarding vertical transmission, placental infection and fetal manifestations for Oropouche virus (OROV), Zika virus (ZIKV), West Nile virus (WNV), and Venezuelan equine encephalitis (VEE) virus infections. Findings are based on published human data and are intended to highlight similarities and differences among congenital arboviral syndromes [2,8,9,10,11,41,45,49,52,53,54,55,56,57,58].

**Table 2:** Recommended testing for maternal and congenital OROV infections.

Stage	Specimens	Tests
Maternal acute infection (≤7d)	Maternal blood	RT-PCR
Maternal post-acute infection (>7d)	Maternal serum	IgM, IgG, PRNT when available
Prenatal evaluation	Amniotic fluid (if prenatal ultrasound suggestive of fetal infectious signs)	RT-PCR
At delivery	Maternal blood, placenta, umbilical cord blood, neonatal blood	RT-PCR and serology
Symptomatic neonate	CSF	RT-PCR, IgM
Fetal demise/stillbirth	Placenta, cord, brain, liver, lung, kidney, spleen and other available tissues	RT-PCR, immunohistochemistry, pathology

## Data Availability

No new data were created or analyzed in this study. Data sharing is not applicable to this article.

## Abbreviations

The following abbreviations are used in this manuscript:

BBB	Blood–brain barrier
CDC	Centers for Disease Control and Prevention
CNS	Central nervous system
CSF	Cerebrospinal fluid
ddPCR	Droplet digital polymerase chain reaction
IFN-I	Type I interferon
IgG	Immunoglobulin G
IgM	Immunoglobulin M
MRI	Magnetic resonance imaging
OROV	Oropouche virus
PAHO	Pan American Health Organization
PCR	Polymerase chain reaction
PRNT	Plaque reduction neutralization test
RT-PCR	Reverse-transcription polymerase chain reaction
RT-qPCR	Quantitative reverse-transcription polymerase chain reaction
TORCH	Toxoplasmosis, Other infections, Rubella, Cytomegalovirus, and Herpes simplex
US	Ultrasound
VEE	Venezuelan equine encephalitis
WHO	World Health Organization
